# Zn-Doping Induced Morphological and Electronic Synergy in Co_3_O_4_ Nanorods for High-Performance Ethylbenzene Sensing

**DOI:** 10.3390/molecules31091389

**Published:** 2026-04-23

**Authors:** Songlin Li, Haoling Wang, Peng Li, Pengfei Cheng, Jiajia Cai, Ruizhe Tian, Qunfeng Niu, Li Wang

**Affiliations:** 1 School of Electrical Engineering, Henan University of Technology, 100 Lianhua Street, Zhengzhou 450001, China; 2 Institute for Complexity Science, Henan University of Technology, 100 Lianhua Street, Zhengzhou 450001, China; 3 School of Aerospace Science and Technology, Xidian University, 266 Xifeng Road, Xi’an 710126, China

**Keywords:** Zn-doped Co_3_O_4_ ethylbenzene sensing, metal oxide semiconductor gas sensor, morphology regulation, surface oxygen species, density functional theory (DFT)

## Abstract

In this study, Zn-doped Co_3_O_4_ nanorods and nanosheets with controlled Zn/Co molar ratios were synthesized via a hydrothermal strategy to clarify the respective roles of morphology and elemental doping in ethylbenzene sensing. The gas-sensing performance is strongly influenced by morphology, and the radially oriented nanorod structure significantly enhances sensing response compared with nanosheet structures. Zn doping effectively enhances the gas-sensing performance of Co_3_O_4_. As a result, the optimized Zn-doped nanorod sensor exhibits high sensitivity to ethylbenzene, a low detection limit, rapid response and recovery, and excellent operational stability. Density functional theory calculations reveal that the predominantly exposed facets of the nanorod structure possess stronger adsorption affinity and pronounced charge transfer toward ethylbenzene, providing theoretical support for the morphology-dominated sensing behavior. At the same time, Zn incorporation further adjusts the band structure and surface reactivity. Overall, this work elucidates a morphology-dominated and doping-assisted enhancement mechanism, offering clear design principles for high-performance Co_3_O_4_-based ethylbenzene sensors.

## 1. Introduction

Ethylbenzene is a colorless aromatic hydrocarbon widely used as an industrial solvent and as a key intermediate in the production of styrene, polystyrene, and ABS resin [1,2]. Prolonged inhalation of ethylbenzene vapor poses significant health risks, including neurological damage, hepatic and renal dysfunction, and abnormalities in the hematopoietic system [3,4]. Ethylbenzene (and styrene) exposure levels are positively associated with the prevalence and incidence of type 2 diabetes mellitus [5]. Furthermore, ethylbenzene may also adversely affect the digestive, immune, and reproductive systems [6,7]. Moreover, as a typical volatile organic compound (VOC) with high volatility and flammability [8], ethylbenzene not only contributes to atmospheric pollution but also poses considerable safety hazards during industrial production, storage, and operation [9]. Therefore, the development of gas-sensing technologies capable of achieving highly sensitive [10], selective, and stable detection is urgently required to ensure environmental monitoring and public health protection [11,12].

Metal oxide semiconductor (MOS) gas sensors have attracted extensive attention due to their high sensitivity [13], low cost [14], compact structure [15], and facile fabrication [16]. They also exhibit favorable selectivity and stability toward volatile organic compounds (VOCs) such as ethylbenzene [17,18]. Compared with conventional binary metal oxide semiconductor materials (SnO_2_, Co_3_O_4_, WO_3_, NiO, and ZnO) [19,20,21,22,23,24], Co_3_O_4_ nanostructures exhibit distinct characteristics in gas-sensing applications. Their sensing performance is predominantly governed by morphology [25,26,27,28]. Different nanomorphologies determine charge-carrier transport pathways, gas-diffusion efficiency, and the degree of exposure of surface-active sites. These factors collectively contribute to a significant impact on sensing performance. However, conventional Co_3_O_4_-based sensors still suffer from limited sensitivity [29], selectivity [30], and long-term stability, which restricts their practical applications [31,32]. To address these issues [33,34], element doping has been widely adopted as an effective auxiliary strategy [35,36]. This approach can modulate the electronic structure [37], increase carrier concentration, and enhance surface chemical activity [38,39], thereby improving gas-sensing response [40]. Previous studies have shown that appropriate doping optimizes the electronic structure and reaction kinetics of Co_3_O_4_, thereby enhancing its gas-sensing performance [41]. Cao et al. [42] synthesized Ti-doped Co_3_O_4_ nanoparticles and achieved a response of 65.6 toward 50 ppm toluene at 280 °C with excellent selectivity for aromatic gases. Song et al. [43] fabricated W-doped Co_3_O_4_ yolk–shell microspheres and obtained a response of 16.5 toward 5 ppm acetone at 250 °C with a detection limit of 10 ppb. These results demonstrate that Ti and W doping enhance surface redox activity and promote gas adsorption and surface reactions, thereby improving sensing performance. Zhao et al. [44] further modified the electronic structure and increased the oxygen-vacancy concentration by Mn doping. This strategy increased the response to 100 ppm acetone to 46.7 and reduced the detection limit to 0.75 ppb while significantly lowering the reaction activation energy. However, most existing studies focus on typical target gases. Systematic investigations of dopant-assisted enhancement for ethylbenzene and other aromatic VOC sensing under morphology-dominated conditions remain limited. Therefore, developing controllable synthesis strategies for Co_3_O_4_ nanostructures with optimized morphologies is essential for environmental monitoring and industrial VOC detection.

Herein, Zn-doped Co_3_O_4_ nanorods and nanosheets were synthesized via a hydrothermal method. The effects of morphology and Zn doping on the microstructure, surface active oxygen species, and gas-sensing performance were systematically investigated. The enhanced sensing performance is attributed to the radially oriented nanorod architecture, which shortens carrier transport pathways and facilitates gas diffusion, thereby improving the sensing response compared with nanosheet structures. This difference reflects an overall trend rather than an occasional measurement result. Zn doping effectively modulates the electronic structure of Co_3_O_4_, optimizes the Co^3+^/Co^2+^ ratio, and enhances surface-adsorbed oxygen species, thereby promoting gas–solid interfacial reactions and improving gas-sensing performance. DFT calculations show that the exposed facets of nanorods exhibit stronger adsorption toward ethylbenzene with pronounced charge transfer. This finding provides theoretical support for the morphology-dominated gas-sensing performance. In addition, Zn doping modulates the band structure and enhances surface reaction activity, further improving overall gas-sensing performance. These experimental and theoretical results establish a direct correlation between structural characteristics and sensing behavior, providing guidance for the design of high-performance nanorod-based ethylbenzene sensors.

## 2. Results and Discussion

### 2.1. Morphology and Structural Properties

**Figure 1:**
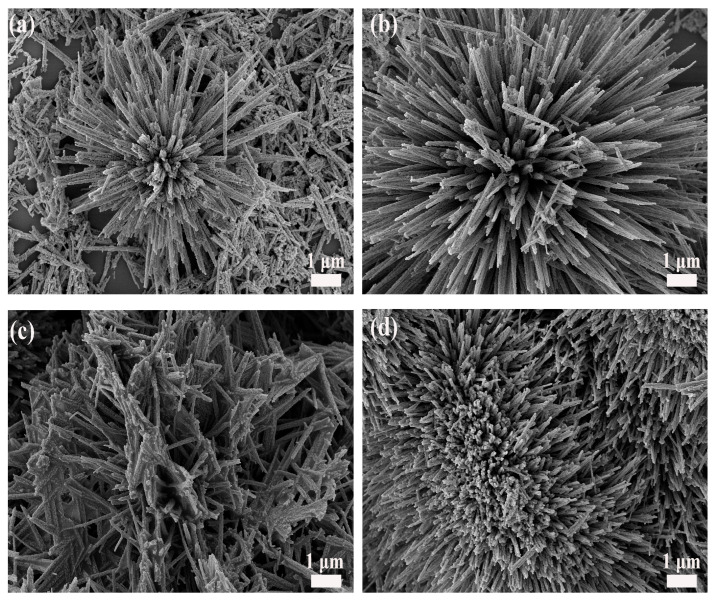
SEM images of Co_3_O_4_ NRs with (**a**) 0 at% Zn, (**b**) 1 at% Zn, (**c**) 2 at% Zn, and (**d**) 3 at% Zn.

**Figure 2:**
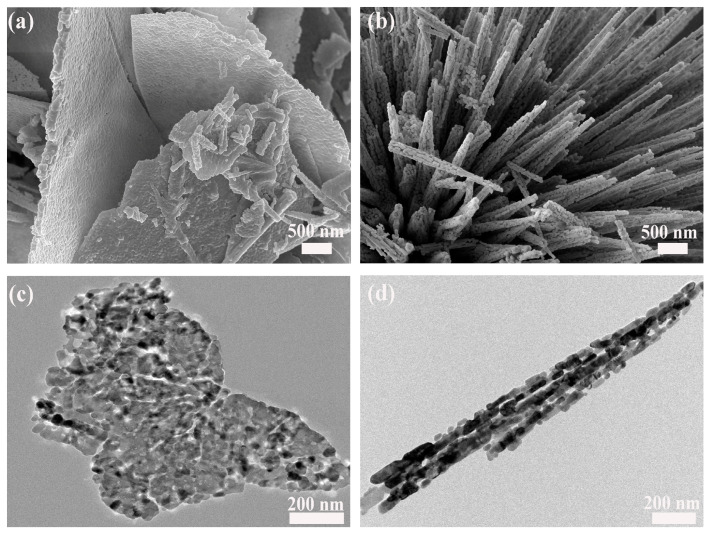
SEM and HRTEM images of 1 at% Zn-doped Co_3_O_4_: (**a**) NSs (SEM), (**b**) NRs (SEM), (**c**) NSs (HRTEM), and (**d**) NRs (HRTEM).

Scanning electron microscopy (SEM) was used to examine the morphological evolution of Co_3_O_4_ as a function of the Zn/Co ratio. As shown in Figure 1a, the undoped sample displays a uniform array of nanorods with relatively smooth surfaces. After introducing 1 at% Zn (Figure 1b), the overall nanorod morphology remains essentially unchanged. Meanwhile, the nanorods become more densely packed with clearer interparticle boundaries, indicating enhanced morphological regularity. At 2–3 at% Zn (Figure 1c,d), the nanorod surfaces become rougher with increased particle aggregation and weakened structural features. This suggests a degradation in morphological definition at higher dopant concentrations. Overall, SEM observations indicate that 1 at% Zn doping yields the most regular and well-defined nanorod morphology. To decouple the effects of morphology from doping, a sample with a distinct two-dimensional structure was synthesized under modified conditions. The resulting 1 at% Zn-doped Co_3_O_4_ nanosheets (Figure 2a) self-assemble into flower-like architectures, presenting a clear morphological contrast to the one-dimensional nanorods (Figure 2b).

**Figure 3:**
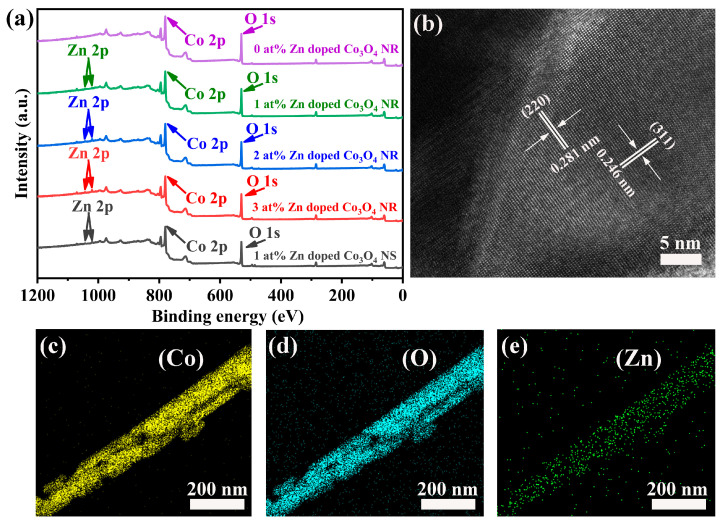
(**a**) XPS survey spectra of Zn-doped Co_3_O_4_ nanorods with Zn/Co ratios of 0%, 1%, 2%, and 3%, and of Zn-doped Co_3_O_4_ nanosheets with a Zn/Co ratio of 1%; TEM image of 1 at% Zn-doped Co_3_O_4_ NR (**b**); and EDS images of 1 at% Zn-doped Co_3_O_4_ NR (**c**–**e**).

TEM analysis provided in-depth insights into the microstructural features of Zn-doped Co_3_O_4_. Figure 2c and Figure 3d display the distinct morphologies of nanorods and nanosheets, revealing that the nanorod architecture actually consists of numerous short nanorods aggregated into a single entity (Figure 2d). Abundant inter-rod pores are formed between these building blocks. XPS was employed to analyze the surface elemental composition and oxidation states of the samples. XPS survey spectra (Figure 3a) reveal Zn, Co, and O signals in 1–3 at% Zn-doped Co_3_O_4_ nanorods and 1 at% nanosheets, unlike undoped samples. These results indicate the successful introduction of Zn into the material system, with no other impurity elements observed. Figure 3b shows clear lattice fringes with interplanar spacings of 0.246 and 0.281 nm, corresponding to the (311) and (220) planes of spinel Co_3_O_4_, confirming the preserved crystalline framework. Notably, local lattice bending and dislocations are also present, which can be attributed to lattice distortion and microstrain induced by Zn^2+^ incorporation. These microstructural perturbations signify an increased density of crystalline defects, which are critical for modulating surface properties. To further verify the uniform incorporation of Zn, energy-dispersive spectroscopy (EDS) elemental mapping was performed. The results (Figure 3c–e) demonstrate a homogeneous spatial distribution of Zn, Co, and O elements with no signs of phase segregation.

**Figure 4:**
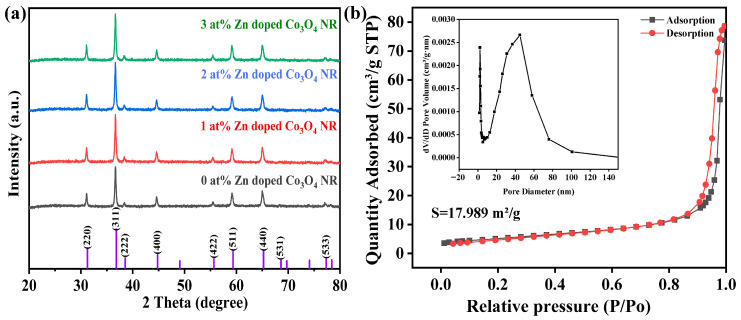
(**a**) XRD patterns of Zn-doped Co_3_O_4_ nanorods with different Zn/Co molar ratios. (**b**) The BET results of 1 at% Zn-doped Co_3_O_4_ were obtained.

The crystallinity and phase characteristics of the nanostructures synthesized with different Zn doping concentrations were further examined by XRD. As shown in Figure 4a, the diffraction peaks of all four samples match well with the standard spinel Co_3_O_4_ phase (PDF#74-2120). The characteristic peaks at 36.5°, 65.4°, and 59.6° can be indexed to the (311), (440), and (511) planes, respectively. The absence of any impurity peaks indicates the high phase purity of the products and confirms the successful incorporation of Zn into the Co_3_O_4_ lattice. Because XRD is less sensitive to subtle local structural variations induced by low-concentration doping, further microscopic analyses were pursued.

The surface area and porosity characteristics were analyzed using nitrogen adsorption–desorption isotherms (Figure 4b). The sample exhibits a typical type IV isotherm with a pronounced hysteresis loop at high relative pressures (P/P_0_ > 0.5), indicating a predominantly mesoporous structure with capillary condensation occurring within the pores. According to the BET method, the specific surface area of the 1 at% Zn–Co_3_O_4_ nanorods was calculated to be 17.989 m^2^/g, with an average pore diameter of 30.4 nm determined by the BJH method from the desorption branch. This accessible pore network, consistent with the observed nanorod morphology, is expected to facilitate mass transport by providing efficient pathways for gas diffusion and active site accessibility. The pore size distribution curve further confirms the presence of a narrow mesopore distribution centered around 30 nm, which is beneficial for rapid analyte penetration. In comparison, the distinct morphology of the nanosheets suggests possible variations in their effective surface area and pore connectivity, potentially leading to different mass transport kinetics and surface reactivity.

**Figure 5:**
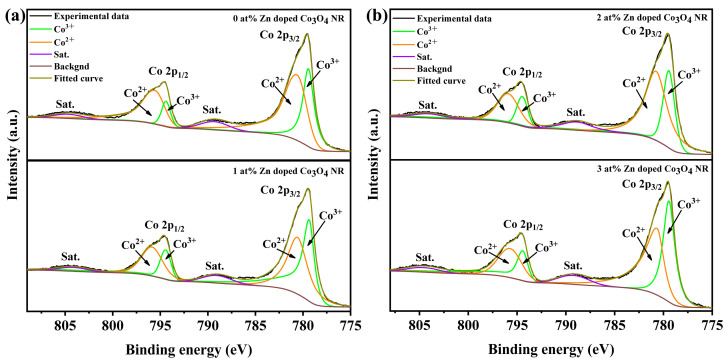
(**a**,**b**) Co 2p XPS spectra of x-Zn-Co_3_O_4_ NRs (x = 0 at%, 1 at%, 2 at%, 3 at%).

Figure 5a,b show the high-resolution Co 2p spectra of 0–3 at% Zn-doped Co_3_O_4_ nanorods. The peaks at approximately 779.7 eV and 794.6 eV correspond to Co 2p_3_/_2_ and Co 2p_1_/_2_, respectively, indicating the coexistence of Co^3+^ and Co^2+^. Quantitative analysis reveals that the Co^3+^/Co^2+^ ratio drops from 1.86 in pristine Co_3_O_4_ to 0.56 at 1 at% Zn doping, then increases at higher Zn levels, indicating preferential Zn^2+^ substitution at Co^3+^ sites with charge compensation.

**Figure 6:**
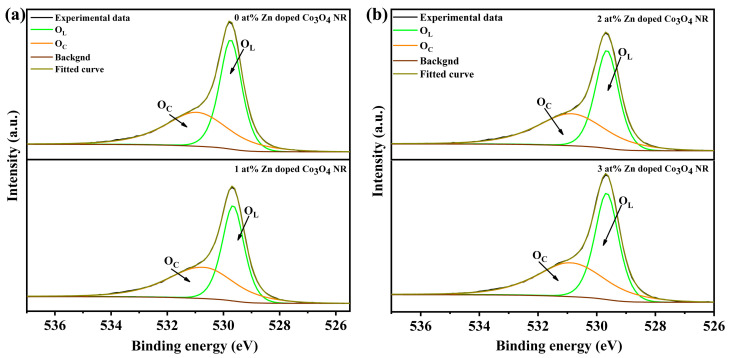
(**a**,**b**) O 1s XPS spectra of x-Zn-Co_3_O_4_ NRs (x = 0 at%, 1 at%, 2 at%, 3 at%).

**Figure 7:**
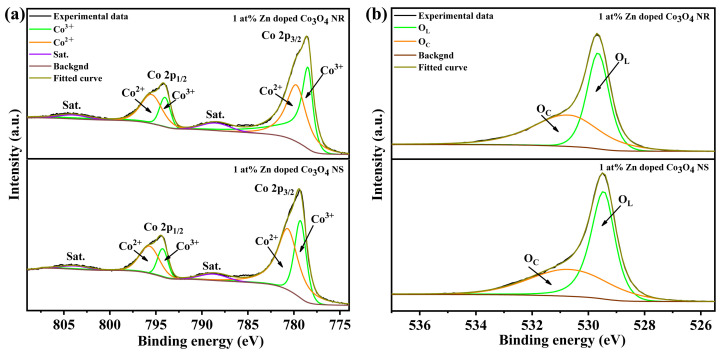
(**a**) Co 2p XPS spectra of 1 at% Zn-Co_3_O_4_ NRs and NS; (**b**) O 1s XPS spectra of 1 at% Zn-Co_3_O_4_ NRs and NS.

To further clarify the surface chemical environment, the O 1s spectra were analyzed (Figure 6a,b). The spectra can be deconvoluted into lattice oxygen (O_L_, ~529.6 eV) and chemisorbed oxygen or oxygen vacancy-related species (O_C_, ~531.2 eV). The O_C_/O_L_ ratio reaches a maximum of 1.08 at 1 at% Zn doping, indicating the most favorable condition for generating surface-active oxygen species. Furthermore, the influence of morphology was evaluated by comparing the 1 at% Zn-doped nanosheets and nanorod. Although both samples exhibit similar Co^3+^/Co^2+^ ratios, the nanosheets show a markedly lower O_C_/O_L_ ratio, suggesting a lower density of surface-active oxygen species. Figure 7a,b show the Co 2p XPS and O 1s spectra of 1 at% Zn-Co_3_O_4_ nanorods and nanosheets. These results indicate that Zn doping alters the relative content of Co^3+^ and Co^2+^ in the Co_3_O_4_ lattice through a substitution mechanism, which serves as a core mechanism for enhancing the gas-sensing performance. The corresponding quantitative results are summarized in Table 1.

### 2.2. Gas-Sensing Performance

**Figure 8:**
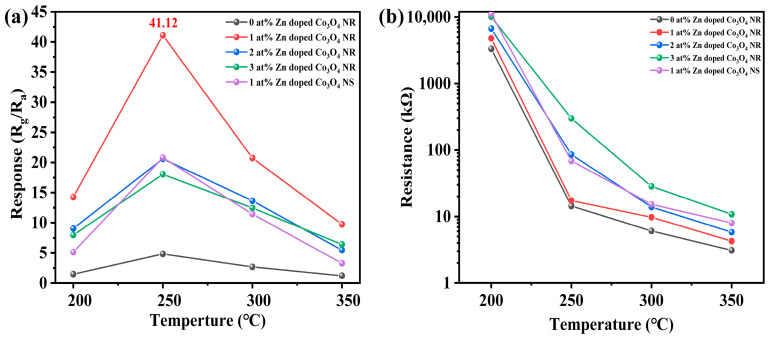
(**a**) Responses of x-Zn-Co_3_O_4_ NRs (x = 0, 1, 2, 3) and Zn-Co_3_O_4_ NS to 100 ppm ethylbenzene at different operating temperatures. (**b**) Initial resistance (R_a_) of all sensors in air at various operating temperatures.

To elucidate the effects of Zn doping level and structural morphology on gas-sensing performance, the sensing behavior of Co_3_O_4_-based sensors toward ethylbenzene was systematically investigated. During the experimental process, different currents were applied to the internal heating resistor of the sensor according to Equation (10) to precisely control the operating temperature. As shown in Figure 8a, the responses of all sensors to 100 ppm ethylbenzene were evaluated over a temperature range of 200–350 °C, a commonly used range for metal oxide semiconductor gas sensors. All samples exhibited a similar temperature-dependent response trend, characterized by an initial increase with rising temperature, followed by a gradual decrease at higher temperatures. This “volcano-type” response profile indicates an optimal operating temperature of approximately 250 °C for all sensors. At temperatures below this value, insufficient thermal energy limits activation of surface reactions between ethylbenzene molecules and chemisorbed oxygen species, resulting in relatively low sensing responses. As temperatures rise, enhanced molecular activation facilitates effective adsorption and oxidation on the sensor surface, leading to a rapid increase in response. However, when the temperature exceeds the optimal point, the desorption rate of gas molecules and surface oxygen species becomes dominant, thereby reducing surface coverage and leading to a decline in sensing response. Among all tested samples, the 1 at% Zn-doped Co_3_O_4_ nanorods consistently showed the highest response across the entire temperature range, reaching a maximum of 41.12 at 250 °C. This value was markedly higher than that of pristine Co_3_O_4_ nanorods (4.79), nanorods with higher Zn doping levels, and the 1 at% Zn-doped nanosheets. These results demonstrate that an optimal Zn doping level together with the nanorod morphology significantly enhances ethylbenzene sensing, making the 1 at% Zn-doped Co_3_O_4_ nanorods the best candidate for further evaluation.

**Figure 9:**
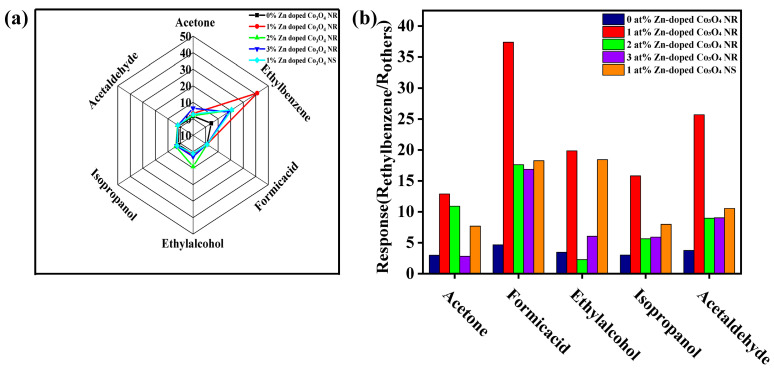
(**a**) Sensor responses to 100 ppm various gases at 250 °C. (**b**) Response ratios to 100 ppm ethylbenzene, ethanol, acetone, isopropanol, acetaldehyde, and formic acid at 250 °C.

The intrinsic electrical properties and selectivity characteristics of the sensors were further analyzed to clarify the origin of the enhanced sensing performance. Figure 8b shows the initial resistance (R_a_) of all sensors measured in air at different operating temperatures. For all samples, the resistance decreased monotonically with increasing temperature, reflecting typical semiconducting behavior. At the same operating temperature, Zn-doped Co_3_O_4_ nanorod sensors exhibited higher initial resistance as the Zn content increased. This trend can be attributed to the combined effects of reduced grain size and increased potential barrier height at grain boundaries induced by Zn doping, which suppresses carrier transport and increases resistance. Selectivity tests were conducted by comparing the responses of sensors with different Zn doping levels toward 100 ppm ethylbenzene, ethanol, acetone, isopropanol, acetaldehyde, and formic acid at their respective optimal operating temperatures. As illustrated in the radar plot in Figure 9a, the 1 at% Zn-doped Co_3_O_4_ sensor exhibited the highest response toward ethylbenzene, significantly exceeding its responses to other interfering gases. The measurements were performed at a relative humidity of 40%, which represents a moderate environmental condition. It is generally recognized that water molecules can compete with oxygen adsorption and alter the surface reaction kinetics. The stable sensing behavior observed under this humidity level indicates that the sensor maintains reliable performance in the presence of ambient moisture. Furthermore, the response ratios (S_ethybenzene_/S_others_) summarized in Figure 9b indicate that the 1 at% Zn-doped sensing performance of the Co_3_O_4_-based sensor, synergistically regulated by the sensor, exhibited the highest selectivity among all samples. Collectively, these results demonstrate that appropriate Zn doping effectively enhances ethylbenzene’s electrical properties, surface chemistry, and gas-discrimination capability.

**Figure 10:**
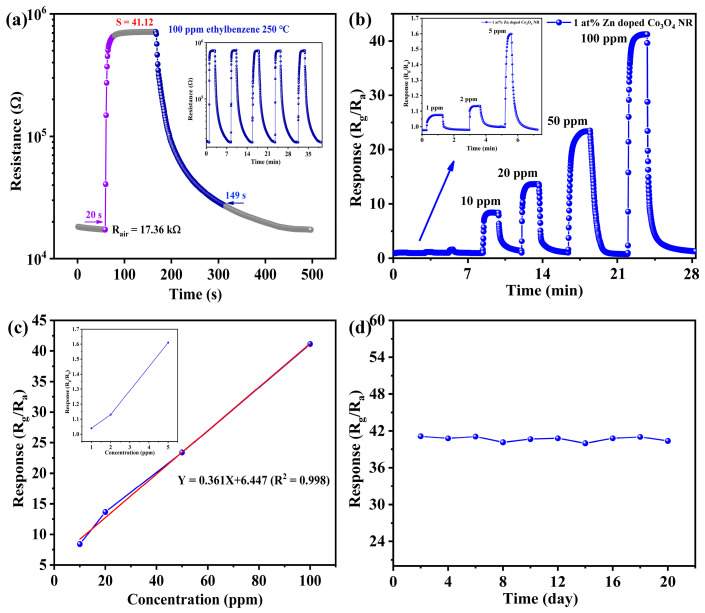
(**a**) Response–recovery curves of 1 at% Zn-doped Co_3_O_4_ nanorod sensor to 100 ppm ethylbenzene at 250 °C (ambient ~25 °C, S represents the response value.). (**b**) Dynamic response–recovery to 1–100 ppm (inset: 1–5 ppm). (**c**) Response and calibration plot (The red line represents the linear fit.). (**d**) 20-day stability.

Following identification of the optimal sensor, its dynamic sensing characteristics were examined in detail to evaluate response speed, reversibility, and short-term operational stability. Figure 10a shows the dynamic transient response of the 1 at% Zn-doped Co_3_O_4_ sensor to 100 ppm ethylbenzene at an operating temperature of 250 °C. Upon introduction of ethylbenzene, the sensor resistance increased sharply, indicating a rapid surface reaction between ethylbenzene molecules and chemisorbed oxygen species. When the target gas was removed and the sensor was exposed to ambient air, the resistance promptly returned to its initial value, demonstrating good reversibility of the sensing process. The response and recovery times were determined to be 20 s and 149 s, respectively, indicating fast surface reaction kinetics and efficient desorption. The inset of Figure 10a shows five consecutive sensing cycles, during which the response curves overlapped almost completely. This result confirms excellent short-term repeatability and signal stability, indicating that the sensing performance is highly reproducible during repeated exposure to ethylbenzene. The rapid and stable dynamic response can be attributed to the synergistic effects of Zn-induced surface electronic modulation and the one-dimensional nanorod architecture. Together, they promote efficient gas diffusion, rapid charge transfer, and stable surface reactions. These characteristics are essential for continuous and real-time monitoring of ethylbenzene in practical applications.

In addition to dynamic response behavior, the concentration-dependent sensing performance of the optimized sensor was further evaluated to assess its quantitative detection capability. As shown in Figure 10b, the response of the 1 at% Zn-doped Co_3_O_4_ sensor increased progressively with ethylbenzene concentration in the range of 5–100 ppm. Meanwhile, it maintained stable and reversible response–recovery characteristics across the entire concentration range. This monotonic increase in response demonstrates that the sensor can reliably distinguish different ethylbenzene concentrations. Linear fitting analysis revealed a strong linear relationship between sensing response and gas concentration. This relationship can be expressed as Y = 0.361X + 6.447, with a high correlation coefficient (R^2^ = 0.998) (Figure 10c). These results indicate excellent linearity and reliable quantitative performance. Based on the signal-to-noise ratio criterion, the detection limit was calculated using the following equations:

(1) $$RMS_{\mathrm{noise}}=\sqrt{\frac{S^{2}}{N}}$$

(2) $$DL=3\times \frac{RMS_{\mathrm{noise}}}{\mathrm{slope}}$$

Here, *S* represents the standard deviation of the fitted curve, *N* denotes the total number of data points, and slope corresponds to the slope of the calibration curve. The factor of 3 indicates that a signal-to-noise ratio of S/N=3 was adopted as the detection limit criterion.

The calculated detection limit was as low as 1 ppm, demonstrating the optimized sensor’s capability to detect trace levels of ethylbenzene. To further assess long-term reliability, the sensor was continuously tested toward 100 ppm ethylbenzene for 20 days under stable operating conditions (at a sensor operating temperature of 250 °C, while the ambient temperature was maintained at 25 ± 1 °C), as shown in. Throughout the entire testing period, the response retention remained consistently above 90%, indicating excellent long-term stability and operational durability. In addition to the long-term response stability, the power consumption and thermal stability of the sensor are also critical for practical applications. In this work, the sensor operates at an optimal temperature of 250 °C, which is maintained by an integrated resistive heating element. Although elevated operating temperatures are typical for metal oxide semiconductor sensors, the relatively moderate working temperature in this study helps limit energy consumption compared with high-temperature (>300 °C) systems reported in the literature. This stable performance suggests that the sensor maintains robust structural integrity and surface chemical activity during prolonged operation, a critical requirement for real-world gas-monitoring applications.

From a mechanistic perspective, the stable response retention above 90% over 20 days indicates that the Zn-doped Co_3_O_4_ nanorods possess excellent thermal stability under continuous heating conditions (Figure 10d). This stability can be attributed to the robust spinel crystal structure and the strong interaction between Zn dopants and the Co_3_O_4_ lattice, which effectively suppress structural degradation and grain coarsening during long-term operation. In addition, the one-dimensional nanorod architecture provides structural integrity and reduces thermal stress accumulation, further contributing to the durability of the sensing material under repeated heating–cooling cycles. This stable performance suggests that the sensor maintains robust structural integrity and surface chemical activity during prolonged operation, a critical requirement for real-world gas-monitoring applications. To further highlight the performance advantages of the 1 at% Zn-doped Co_3_O_4_ nanorods, a comprehensive comparison of the operating temperature, response magnitude, and detection limit with other recently reported Co_3_O_4_-based sensors is summarized in Table 2.

### 2.3. DFT Calculations

To elucidate the microscopic adsorption behavior of ethylbenzene on Co_3_O_4_ surfaces, density functional theory (DFT) calculations were performed on the predominantly exposed (110) and (111) facets. The (110) facet corresponds to the characteristic surface of nanorod morphologies, whereas the (111) facet represents the typical exposed surface of nanosheet structures. These results provide a rational explanation for the experimentally observed morphology-dependent sensing performance. The adsorption energy was calculated as:

(3) $$\Delta E=E_{\mathrm{total}}-E_{\mathrm{slab}}-E_{\mathrm{adsorbate}}$$

where *E*_total_, *E*_slab_, and *E*_adsorbate_ represent the total energies of the adsorption system, the clean surface slab, and the isolated gas molecule, respectively. To ensure a consistent and meaningful comparison between morphologies, all calculations were performed on 1 at% Zn-doped Co_3_O_4_ surfaces.

**Figure 11:**
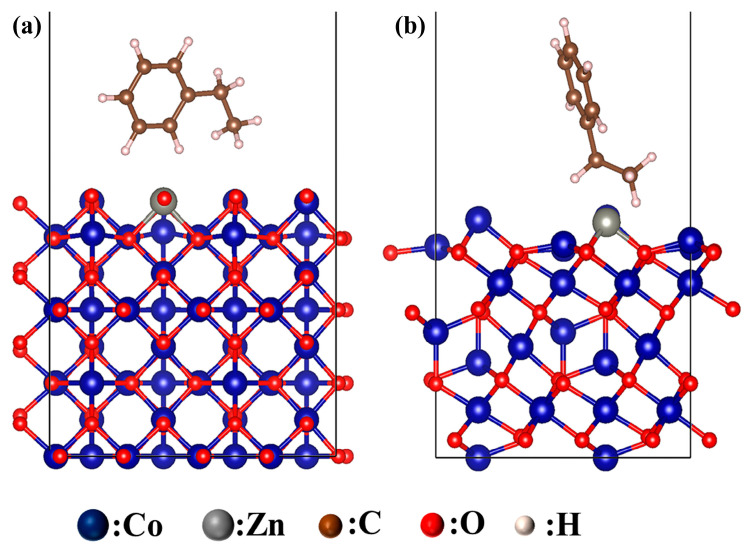
DFT-optimized adsorption configurations of ethylbenzene on (**a**) Zn-doped Co_3_O_4_ (110) nanorod surface and (**b**) Zn-doped Co_3_O_4_ (111) nanosheet surface.

Figure 11 shows the optimized adsorption configurations of ethylbenzene on the (110) facet of Zn-doped Co_3_O_4_ nanorods and the (111) facet of Zn-doped Co_3_O_4_ nanosheets. Table 2 summarizes the corresponding adsorption energies (*E*_ads_). The calculated adsorption energy on the (110) facet of the 1 at% Zn-doped Co_3_O_4_ nanorods is −2.30 eV, which is significantly lower than that on the (111) facet of the nanosheets (−0.54 eV). This pronounced difference indicates that the (110) facet exhibits a much stronger adsorption affinity and greater adsorption stability toward ethylbenzene. As a result, it provides more favorable thermodynamic conditions for molecular adsorption and subsequent surface reactions.

In addition to adsorption strength, interfacial charge transfer plays a critical role in resistance modulation during gas sensing. Charge-transfer analysis shows that ethylbenzene adsorption on the Co_3_O_4_ nanorod (110) facet induces a charge transfer of −0.026386 e, approximately 4.5 times larger than that on the nanosheet (111) facet (−0.005898 e) (Table 3). This pronounced interfacial electron exchange enhances modulation of the surface carrier concentration, thereby amplifying the sensor’s resistance response. Overall, these DFT results provide atomic-scale evidence for the experimentally observed performance disparity. Co_3_O_4_ nanorods predominantly exposing the (110) facet exhibit superior ethylbenzene sensing performance compared with nanosheets mainly exposing the (111) facet. This enhancement arises from stronger adsorption and greater charge transfer.

### 2.4. Gas Sensing Mechanism

**Figure 12:**
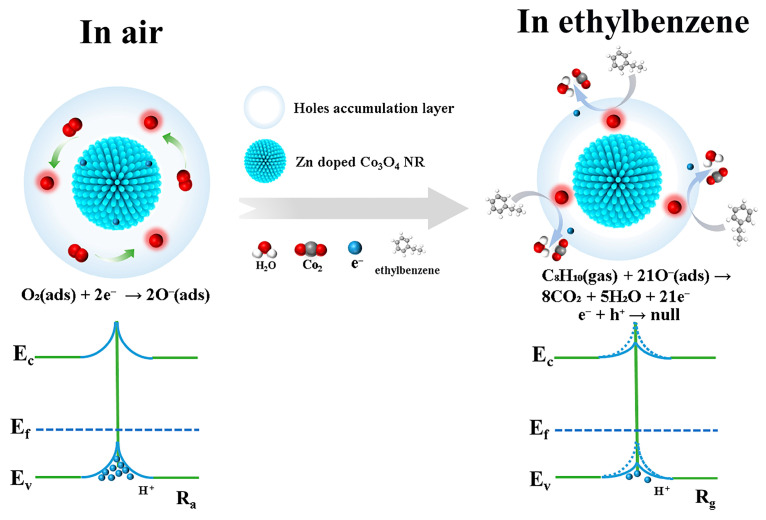
Schematic of ethylbenzene sensing mechanism in Co_3_O_4_ nanorods.

The gas-sensing mechanism of metal oxide semiconductors is widely recognized as governed by adsorption–desorption processes on their active surfaces. In p-type semiconductors such as Zn-doped Co_3_O_4_, holes are the majority charge carriers and dominate electrical conduction (Figure 12). When the sensor is exposed to air, oxygen molecules adsorb onto the surface and capture electrons from the conduction band, forming reactive oxygen species such as O_2_^−^, O^−^, and O^2−^. This electron-withdrawal process leads to the accumulation of holes near the surface, creating a hole-accumulation layer that determines the sensor’s baseline resistance. The specific oxygen species participating in the sensing process depends strongly on the operating temperature. At temperatures below 100 °C, O_2_^−^ species are predominant. With increasing temperature (100–300 °C), O^−^ species become the most active. Above 300 °C, O^2−^ species contribute more significantly to surface reactions. At an operating temperature of 250 °C employed in this work, O^−^ species are considered the primary reactive oxygen ions responsible for ethylbenzene sensing. These temperature-dependent surface reactions are illustrated by Equations (4)–(7):

(4) $$O_{2}(\mathrm{gas})\to O_{2}(\mathrm{ads})$$

(5) $$O_{2}(\mathrm{ads})+e^{-}\to O_{2}^{-}(\mathrm{ads})\;\;\;(T\leq {100\;}^{{}^{\circ}}C)$$

(6) $$O_{2}^{-}(\mathrm{ads})+e^{-}\to 2O^{-}(\mathrm{ads})\;\;\;({100\;}^{{}^{\circ}}C<T\leq {300\;}^{{}^{\circ}}C)$$

(7) $$O_{2}(\mathrm{ads})+2e^{-}\to 2O^{-}(\mathrm{ads})\;\;\;(T>{300\;}^{{}^{\circ}}C)$$

At an operating temperature of 250 °C, the gas-sensing process is dominated by the surface reaction between ethylbenzene molecules and adsorbed O^−^ species. Upon exposure to ethylbenzene, the target gas reacts with surface O^−^ ions, producing CO_2_ and H_2_O while releasing electrons, as described by the reaction:

(8) $$C_{8}H_{10}+21O^{-}\to 8{\mathrm{CO}}_{2}+5H_{2}O+21e^{-}$$

(9) $$e^{-}+h^{+}\to \mathrm{null}$$

Released electrons recombine with holes in the hole-accumulation layer (*e*^−^ + *h*^+^ → null), decreasing hole concentration and increasing resistance. Upon air exposure, oxygen re-adsorbs, rebuilding the hole-accumulation layer and restoring initial resistance. This reversible modulation of hole concentration during ethylbenzene/air cycling produces characteristic resistance changes. The stable, repeatable response confirms the reversible adsorption–reaction–desorption process on Zn-doped Co_3_O_4_ surface.

The superior ethylbenzene-sensing performance of the 1 at% Zn-doped Co_3_O_4_ nanorods can be attributed to the synergistic effects of optimized Zn doping and favorable nanorod morphology. First, the results demonstrate that the gas-sensing performance is predominantly governed by morphology. Compared with nanosheets, the nanorods exhibit a lower Co^3+^/Co^2+^ ratio (0.56) and a higher O_C/O_L ratio (1.08), indicating a greater abundance of reactive chemisorbed oxygen species available for surface reactions. Second, the one-dimensional nanorod architecture provides efficient gas-diffusion pathways and exposes more accessible active sites, facilitating rapid transport and effective interaction of ethylbenzene molecules with reactive oxygen species. In contrast, the thicker, densely stacked nanosheet structure restricts gas diffusion and limits the utilization of active surface sites, thereby reducing sensing efficiency. Consequently, Zn doping enhances the surface chemistry, while the nanorod morphology improves mass-transport characteristics. Together, these advantages lead to significantly increased sensitivity, faster response, and superior selectivity of the 1 at% Zn-doped Co_3_O_4_ nanorod sensor toward ethylbenzene.

## 3. Materials and Methods

### 3.1. Materials

Cobalt nitrate (Co(NO_3_)_2_·6H_2_O, 99%) was purchased from China National Pharmaceutical Group Chemical Reagent Co., Ltd (Shanghai, China). Ammonium fluoride (NH_4_F, 99%), urea (CO(NH_2_)_2_, 99%), sodium citrate (C_6_H_5_Na_3_O_7_·2H_2_O, 99%), and zinc nitrate (Zn(NO_3_)_2_·6H_2_O, 99%) were obtained from Aladdin Reagent Co., Ltd (Shanghai, China). for material synthesis. The target and interference gases used for gas-sensing performance evaluation, including ethylbenzene (99.8%), acetaldehyde (99%), acetone (99.5%), isopropanol (99.5%), ethanol (99.7%), and formic acid (85%), were also supplied by Aladdin Reagent Co., Ltd. (Shanghai, China). All chemicals were of analytical grade and used as received without further purification. Anhydrous ethanol and deionized water were employed as solvents throughout the material preparation and device fabrication processes.

### 3.2. Sample Synthesis

**Figure 13:**
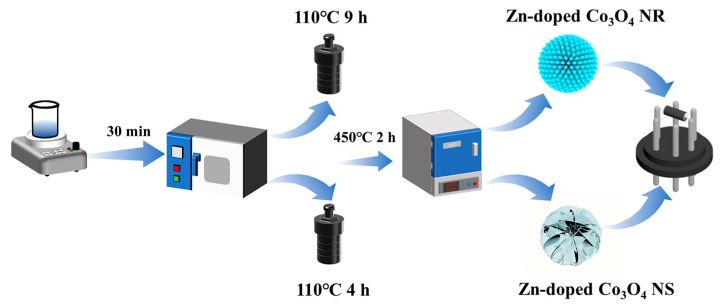
Schematic illustration of the synthesis of two distinct Zn-doped Co_3_O_4_ nanostructures.

Urea (10 mmol), ammonium fluoride (8 mmol), cobalt nitrate (2 mmol), varying amounts of zinc nitrate (Zn (NO_3_) _2_·6H_2_O, corresponding to Zn/Co molar ratios of 0 at%, 1 at%, 2 at%, and 3 at%), and sodium citrate (0.03 mmol) were dissolved in 36 mL of deionized (DI) water and stirred at room temperature for 30 min. The resulting pink solution was transferred to a 40 mL Teflon-lined autoclave and heated at 110 °C for 9 h to obtain nanorod precursors. In contrast, a 4 h reaction produced nanosheet precursors. After cooling to room temperature, the products were collected, washed three times with DI water and ethanol, and dried in air at 80 °C for 6 h. Subsequently, the dried samples were calcined in air at 450 °C for 2 h, yielding Zn-doped Co_3_O_4_ nanorods (NR) and nanosheets (NS), respectively. The synthesis procedures for the different Zn-doped Co_3_O_4_ morphologies are summarized in Figure 13.

### 3.3. Characterization

The crystal phases of the synthesized Zn-doped Co_3_O_4_ samples were characterized by X-ray diffraction (XRD, Rigaku Ultima IV, Rigaku Corporation, Tokyo, Japan) over a 20–80° range using Cu Kα radiation (λ = 1.5418 Å). The surface morphology and microstructure were examined by field-emission scanning electron microscopy (FE-SEM, Hitachi S-4800, Tokyo, Japan) equipped with an energy-dispersive X-ray spectrometer (EDS, Oxford X-MaxN, Oxford, UK), and further analyzed by transmission electron microscopy (TEM, Tecnai F20, Hillsboro, OR, USA) and high-resolution TEM (HRTEM, Hillsboro, OR, USA). Brunauer–Emmett–Teller (BET) specific surface area and pore-size distribution were evaluated using N_2_ adsorption–desorption isotherms (Micromeritics ASAP 2460, Norcross, GA, USA). In addition, the elemental composition and oxidation states were determined by X-ray photoelectron spectroscopy (XPS, Thermo ESCALAB 250Xi, Waltham, MA, USA), providing essential information for subsequent performance analysis.

### 3.4. Sensor Fabrication and Gas Sensing Measurements

The prepared samples were mixed with an appropriate amount of distilled water to form a homogeneous paste, which was then brushed onto the surface of alumina ceramic tubes to form a uniform sensing film. Ring-shaped gold electrodes were deposited on both ends of the ceramic tube and electrically connected with platinum wires. Subsequently, a miniature resistive heating wire was inserted into the ceramic tube to regulate the sensor’s operating temperature.

The operating temperature (*T*, °C) of the sensor was calibrated as a function of the heating current (*I*, mA) using the following relationship:

(10) T=2.5×I−50

where 2.5 is the temperature–current conversion coefficient (°C·mA^−1^) and 50 is the temperature correction term (°C).

Prior to gas-sensing measurements, sensors based on Zn-doped Co_3_O_4_ NRs (0–3%) and Zn-doped Co_3_O_4_ NSs (1%) were electrically aged on a circuit board for 72 h to stabilize the interfacial contact between the sensing film and the ceramic substrate, thereby improving the device’s operational stability. Gas-sensing performance was evaluated using a static gas testing system under controlled environmental conditions. The ambient temperature was maintained at 25 ± 1 °C. All measurements were conducted in two 1 L sealed chambers, in which the sensors were alternately exposed to dry air and the target gas. The sensor’s real-time resistance variation was monitored with a digital multimeter (Fluke 8846A, Everett, WA, USA) to evaluate the sensing response and stability.

The target gas concentration was achieved by injecting a calculated volume of the appropriate liquid reagent into the test chamber using a microsyringe. This was calculated based on the ideal gas relationship, expressed as:

(11) $$C=\frac{V_{m}\cdot \rho \cdot w\cdot V_{1}}{M\cdot V_{2}}\times 10^{3}$$

where *C* is the target gas concentration (typically in ppm, defined as μmol·mol^−1^), *V*_1_ (μL) and *V*_2_ (1 L) are the volumes of the liquid to be measured and the test chamber, respectively, *M* is the molar moss of the target gas (g·mol^−1^), *V_m_* is the molar volume of an ideal gas at standard temperature and pressure (22.4 L·mol^−1^), *ρ* is the density of the liquid reagent (g·cm^−3^), and *w* is the mass fraction (purity) of the liquid reagent (wt%).

Response time and recovery time are defined as the time required to achieve 90% of the total resistance change separately in the response and recovery process. The sensor response (R) was defined as:

(12) $$R=\frac{R_{g}}{R_{a}}$$

where R_g_ and R_a_ represent the sensor resistance in the presence of the target gas and in air, respectively, the response and recovery times were defined as the durations required for the sensor resistance to reach 90% of the total resistance change during adsorption and desorption, respectively.

### 3.5. Calculation Detail

The theoretical computations were carried out using the first-principles simulation package VASP (Vienna Ab initio Simulation Package, vasp.6.1.0) [49,50]. The exchange–correlation interactions were described using the GGA-PBE functional [51,52], together with PAW-PBE pseudopotentials [53,54]. To address the strong electron–electron interactions associated with the 3d orbitals of Co and Zn, the GGA + U approach was adopted. The effective Hubbard parameters were set to Ueff(Co) = 3 eV [55] and Ueff(Zn) = 6 eV, in accordance with previous reports. The plane-wave cutoff energy was fixed at 450 eV [56]. The convergence thresholds for self-consistent field (SCF) calculations and ionic relaxation were set to 1 × 10^−5^ eV for electronic energy and 0.02 eV/Å for atomic forces, respectively. The Brillouin zone sampling was generated using VASPKIT with a K-spacing parameter of 0.03. To account for van der Waals interactions between gas molecules and the slab surface, the DFT-D3 correction method proposed by Grimme with zero damping was applied.

The (110) and (111) surfaces were constructed from the primitive cell (a = b = c = 5.729 Å; α = β = γ = 60°), and expanded into a 2 × 2 × 2 supercell. A vacuum layer of 15 Å was introduced along the Z direction to minimize interactions between periodic images perpendicular to the surface. During structural optimization, atoms in the lower half of the slab along the Z axis were fixed to mimic bulk characteristics, while the upper half was fully relaxed to represent surface behavior. Considering the structural complexity of Co_3_O_4_ and the asymmetry between the top and bottom surfaces in the slab model, dipole corrections were applied in the adsorption calculations.

## 4. Conclusions

In summary, Co_3_O_4_ nanorods and nanosheets with different Zn doping concentrations were successfully synthesized via a hydrothermal method. Zn doping effectively modulates the Co^3+^/Co^2+^ ratio and increases the concentration of surface-active oxygen species, as confirmed by XPS analysis. Among all samples, the nanorod-based sensor doped with 1 at% Zn exhibited the highest response of 41.12 to 100 ppm ethylbenzene at 250 °C under 40% relative humidity, together with excellent selectivity and long-term stability. Moreover, the sensor demonstrates relatively low power consumption due to its moderate operating temperature and exhibits excellent thermal stability under prolonged operation, highlighting its potential for practical and continuous gas-monitoring applications. The effect of humidity was briefly evaluated at 40% RH; however, ambient humidity variations may influence the adsorption–desorption processes of oxygen species and target gas molecules, thereby affecting sensor response. Further systematic investigations under a wider range of relative humidity conditions are necessary to fully evaluate the practical applicability of the sensor. From the perspective of intrinsic material modulation, Zn doping optimizes the relative abundance of Co^3+^ species and promotes efficient interconversion between Co^3+^/Co^2+^ redox sites, thereby facilitating the enrichment of surface-active oxygen species and enhancing electron transport. Moreover, Zn-induced surface chemical modulation reduces the activation energy of oxygen-related reactions, thereby improving the catalytic activity during gas sensing. On this basis, the composition, structure, and physicochemical properties of the 1 at% Zn-doped Co_3_O_4_ nanorod sensor were systematically characterized using multiple structural and surface analysis techniques. The results indicate that the nanorod architecture provides efficient gas-diffusion pathways and exposes abundant active sites, while Zn doping further amplifies these morphology-derived advantages by regulating microstructure and surface chemistry. These findings highlight the strong potential of this material for practical ethylbenzene detection. Overall, this work provides a feasible material design strategy for high-performance Co_3_O_4_-based ethylbenzene sensors.

## Figures and Tables

**Table 1:** The variation in Co valence state (Co^3+^/Co^2+^) and oxygen species ratio (O_C_/O_L_) with Zn doping concentration in NR and NS samples.

Zn Doping Ratio (at%)	Co^3+^/Co^2+^	O_C_/O_L_
0 NR	1.86	0.85
1 NR	0.56	1.08
2 NR	0.8	0.96
3 NR	1.08	0.83
1 NS	0.65	0.74

**Table 2:** Comparison of sensor responses, operational temperature, and LOD towards ethylbenzene.

Sen. Mater.	T (°C)	EB.	Resp.	Det. Limit	Ref.
ZnO-CeO_2_	200 °C	50 ppm	19.9	5 ppm	[45]
Co-C_3_N_4_/ZnO	370 °C	100 ppm	8.5	—	[46]
SnO_2_/V_2_O_5_	270 °C	200 ppm	10.5	0.5 ppm	[47]
Co_3_O_4_-In_2_O_3_	75 °C	125 ppm	19.1	—	[48]
Zn-doped Co_3_O_4_	250 °C	100 ppm	41.12	1 ppm	This work

**Table 3:** Ethylbenzene adsorption on Zn-doped Co_3_O_4_ nanorods (110) and nanosheets (111) was modeled, with adsorption energy (Eads) and charge transfer (Q) calculated.

Site	Zn-Co_3_O_4_ NR	Zn-Co_3_O_4_ NS
Adsorption energy (eV)	−2.3	−0.54
Transferred electron (e)	−0.026	−0.0059

## Data Availability

Dataset available on request from the authors.
